# Atopic dermatitis and the gut-skin axis: a review from dysbiosis to novel targeted therapeutic strategies

**DOI:** 10.3389/fimmu.2026.1895580

**Published:** 2026-07-15

**Authors:** Ruzhen Peng, Xiao Huang, Yindi Bao, Guangwen Luo, Junfen Zeng, Liu Tang

**Affiliations:** 1 Department of Pharmacy, Renmin Hospital of Wuhan University, Wuhan, China; 2 Hubei Key Laboratory of Resources and Chemistry of Chinese Medicine, School of Pharmacy, Hubei University of Chinese Medicine, Wuhan, China; 3 Department of Obstetrics and Gynecology, Renmin Hospital of Wuhan University, Wuhan, Hubei, China; 4 Department of Pharmacy, Affiliated Jinhua Hospital, Zhejiang University School of Medicine, Jinhua, China

**Keywords:** atopic dermatitis, dysbiosis, gut microbiota, immune homeostasis, skin microbiota, targeted therapy

## Abstract

Atopic dermatitis (AD) is a common, chronic, relapsing, inflammatory skin disorder with a complex pathogenesis involving genetic, immune, and environmental factors. Recent advances in the research on the gut-skin axis (GSA) have shed new light on the pathophysiology of AD, highlighting the critical role of gut microbiota dysbiosis in immune regulation and inflammatory responses. This review summarizes gut and skin microbial dysbiosis in AD and its impact on immune homeostasis, elaborates on molecular/cellular mechanisms by which gut–skin axis dysfunction drives AD inflammation, and focuses on targeted therapeutic strategies for gut–skin axis modulation. It outlines the latest research and clinical translation of interventions, including probiotics, prebiotics, postbiotics, dietary adjustments, fecal microbiota transplantation, and microbe/metabolite-targeted biologics, providing a theoretical basis for AD precision management and novel therapeutic development.

## 1 Introduction

Atopic dermatitis (AD) is a heterogeneous, chronic, relapsing inflammatory skin disorder characterized by intense pruritus, eczematous lesions, and profound barrier dysfunction. Its global prevalence has risen steadily in industrialized nations, imposing substantial burdens on quality of life, mental health, and socioeconomic systems (1). The conventional pathogenic paradigm centers on a dyad of intrinsic epidermal barrier defects (often linked to filaggrin mutations) and a dysregulated, type 2-skewed immune response involving IL-4, IL-13, and IL-31 (2). Although this mechanistic model has facilitated the development of targeted biologic agents such as dupilumab, it remains insufficient to fully account for the extensive phenotypic heterogeneity of AD, its association with the atopic march, and its chronic relapsing clinical course. Such unresolved limitations have prompted a conceptual shift toward identifying systemic and environmental regulators that modulate AD onset and progression. Research efforts in this regard have increasingly converged on the gut–skin axis, a bidirectional communication network that connects the gastrointestinal tract and the skin via neural, endocrine, immune and metabolic signaling crosstalk.

Resident microbiota in both the gut and skin serve as central mediators of this interorgan dialogue. Under physiological conditions, microbial communities sustain barrier homeostasis, orchestrate immune maturation, and synthesize metabolites essential for immune balance. In the setting of AD, high-throughput sequencing has consistently confirmed microbial dysbiosis at both mucosal and cutaneous sites, characterized by reduced microbial diversity and disrupted community composition (1, 3). Intestinal dysbiosis in AD is characterized by decreased short-chain fatty acid (SCFA)-producing bacteria (e.g. *Faecalibacterium*, *Roseburia*) and enrichment of pro-inflammatory microorganisms (4). Meanwhile, lesional skin displays decreased microbial diversity alongside dominant colonization of *Staphylococcus aureus (S. aureus)*, which further aggravates cutaneous inflammation through the secretion of toxins and proteases (5).

This parallel gut and skin dysbiosis are mechanistically intertwined with AD pathogenesis. Impaired intestinal barrier integrity may facilitate the translocation of microbial components and dietary antigens, thereby priming persistent systemic inflammation. In contrast, gut-derived SCFAs confer anti-inflammatory effects and reinforce skin barrier function by regulating regulatory T-cell responses and maintaining epithelial integrity (6). Collectively, the gut–skin axis acts as a pivotal systemic regulatory module in AD pathophysiology, providing a plausible mechanistic framework to interpret how diet, antibiotic exposure, psychological stress and environmental factors jointly influence disease initiation and relapse.

Herein, we performed a narrative literature retrieval across PubMed, Web of Science, CNKI and Wanfang Data until April 2026 with the following search terms: “atopic dermatitis”, “gut-skin axis”, “dysbiosis”, “short-chain fatty acids”, “probiotics”, “prebiotics”, “postbiotics”, “fecal microbiota transplantation (FMT)”, and “biologics”. This review systematically summarizes the evidence linking gut–skin axis dysbiosis to AD pathogenesis, elucidates the underlying immunological and metabolic mechanisms, and critically assess current breakthroughs and pending challenges for microbiota-targeted interventions. Discussed interventions include probiotics, prebiotics, postbiotics, FMT, dietary manipulation, biologics and small-molecule drugs, highlighting priorities for clinical translation and precision medicine implementation.

## 2 The vicious cycle between gut and skin dysbiosis and barrier impairment in AD

Accumulating evidence indicates that gut microbiota dysbiosis represents a key pathological hallmark of AD, especially in infants and young children. Such dysbiosis is typically characterized by reduced microbial diversity and altered composition that correlates with disease severity. Consistent clinical observations have confirmed that infants with AD exhibit significantly lower overall gut microbial diversity relative to healthy controls (1, 4). At the taxonomic level, AD-related gut dysbiosis is featured by the depletion of beneficial acidophilic bacterial taxa, including *Bifidobacterium*, *Lactobacillus* and *Enterococcus* (1, 7). Meanwhile, potentially proteolytic and pathobiontic bacteria, such as *Escherichia coli*, *Klebsiella* species, and *Enterobacter* species, are markedly enriched in the gut of AD infants (1). Notably, this microbial imbalance is not restricted to the infant gut alone. Maternal gut microbiota during pregnancy also displays distinct compositional signatures linked to offspring AD susceptibility, which are characterized by reduced alpha-diversity and decreased abundance of SCFA-producing genera including *Faecalibacterium*, *Roseburia* and *Butyricicoccus* (8). Compositional perturbations of gut microbiota further led to impaired microbial metabolic function, most notably the impaired biosynthesis of SCFAs (e.g., butyrate, propionate and acetate). These metabolites exert robust anti-inflammatory effects and play an essential role in maintaining intestinal epithelial barrier integrity (4, 9, 10). Reduced circulating and intestinal SCFA levels have been documented in both human AD cohorts and preclinical animal models (6, 9, 10).

Of particular importance, gut microbial dysbiosis emerges prior to the clinical onset of AD in multiple prospective birth cohort studies (11, 12). Marrs et al. (11) reported that increased abundance of *Clostridium sensu stricto* is correlated with AD onset at 3 and 12 months of age. A longitudinal cohort study further demonstrated that early infancy perturbations in pivotal microbial taxa, including *Streptococcus*, *Clostridium*, and *Akkermansia*, dictate the natural trajectory of AD, enabling differentiation between transient and persistent AD phenotypes (12). A maternal-prenatal study reveals that gestational gut microbiome perturbations predispose offspring to AD by regulating the intrauterine immune microenvironment (4). Furthermore, mendelian randomization studies have provided genetic evidence supporting a causal relationship between specific gut microbial taxa and AD risk, with directionality tests confirming that the causal flow is primarily from gut microbiota to AD rather than the reverse (13). Collectively, these findings support a contributory causal role for gut dysbiosis in AD pathogenesis, consistent with both a permissive role in predisposing individuals to AD and a contributory role in driving disease progression, rather than merely serving as a secondary consequence of cutaneous inflammation. Nevertheless, it should be acknowledged that AD pathogenesis is multifactorial, and microbial alterations may partly reflect genetic predisposition or systemic immune dysregulation. Thus, gut dysbiosis should be viewed as one component within a complex disease network rather than a sole causative agent.

The gut–skin axis is now widely acknowledged to function in a bidirectional manner. Early-life skin barrier impairment, characterized by elevated transepidermal water loss, can reshape gut microbiome composition *via* systemic inflammatory signaling, and experimental studies have validated that cutaneous injury disrupts intestinal microbial profiles and gut immune homeostasis. Thus, instead of a unidirectional gut-to-skin regulatory pathway, the crosstalk between the gut and skin likely forms a self-reinforcing cycle, wherein cutaneous barrier damage and gut microbial dysbiosis mutually exacerbate and amplify one another (14).

In AD, the skin microbiome also undergoes profound dysbiosis, primarily manifested as a collapse in microbial diversity and a pathological dominance of *S. aureus.* This microbial alteration interacts synergistically with impaired skin barrier function to form a self-perpetuating vicious cycle. Compared with healthy skin, lesional skin in AD patients displays markedly decreased microbial richness and is frequently colonized and dominated by *S. aureus*. Its virulence factors, such as δ-toxin, directly disrupt the epidermal barrier and robustly activate immune cells, thereby potentiating type 2 inflammation (15). Such skin dysbiosis is further predisposed by intrinsic epidermal barrier defects, among which filaggrin (*FLG*) mutations are well-established contributors (16, 17). The dysbiotic state is defined by excessive proliferation of pathogenic *S. aureus* alongside depletion of beneficial commensal bacteria, represented by *Staphylococcus epidermidis* (18, 19). A compromised skin barrier provides a favorable niche for *S. aureus* colonization and expansion. In turn, *S. aureus* secretes abundant proteases and toxins that further degrade key barrier structural proteins, including filaggrin and desmoglein-1, and exacerbate cutaneous inflammation (3, 15, 20). This self-sustaining pathogenic cycle arises early in the disease course, correlates with disease severity across distinct AD dermotypes, and ultimately maintains chronic skin inflammation. In this chronic state, skin microbiome dysbiosis acts as a key driver that continuously aggravates barrier impairment and inflammatory cascade (18).

## 3 Immune and metabolic communication in the gut-skin axis

Gut-associated lymphoid tissue (GALT)-mediated immune modulation represents a core mechanistic pathway in the gut-skin axis. The gut-skin axis functions as a dynamic bidirectional communication network, whereby gut microbiota orchestrate systemic and cutaneous immunity and thereby critically contribute to AD pathogenesis. This process hinges on microbial regulation of immune cells within GALT. Certain probiotic strains, such as *Lactobacillus paracasei* and *Bifidobacterium bifidum*, can expand regulatory T cell populations, upregulate skin barrier-related genes, and concurrently suppress pro-inflammatory cytokine production in experimental AD models (21, 22). This gut-initiated immunomodulation exerts systemic impacts: educated immune cells and soluble signaling cytokines enter the bloodstream and remotely reshape the cutaneous immune microenvironment.

Gut microbial metabolites, particularly SCFAs, act as pivotal molecular mediators beyond immune cell priming. Upon systemic circulation, SCFAs reinforce the skin barrier, modulate keratinocyte metabolism, and directly inhibit central inflammatory cascades such as the IL-33/IL-13 signaling pathway (23, 24). These metabolites also boost anti-inflammatory cytokine secretion and sustain regulatory T cell (Treg) function. In parallel, gut microbiota metabolizes dietary tryptophan into bioactive derivatives (e.g., kynurenines) that further participate in immune homeostasis regulation (25). Mechanistically, gut-derived tryptophan catabolites including kynurenine and indole derivatives, enter the circulation, bind aryl hydrocarbon receptor (AHR) on keratinocytes, and drive AHR-ARNT-dependent transcription of barrier genes such as *filaggrin*, thereby preserving epidermal integrity and immune homeostasis (26–30). Additionally, intestinal microbiota dysbiosis-mediated aberrant bile acid metabolism provides a plausible pathway for gut-skin axis signaling transmission in AD pathogenesis (31). Jena et al. (32) demonstrated that specific activation of TGR5 and sphingosine-1-phosphate receptor 2 (S1PR2) by bile acids aggravates Western diet (WD)-exacerbated AD characterized by enhanced Th2 and Th17 immune responses. As a G protein-coupled receptor, TGR5 activation is critically involved in the modulation of pruritus sensation, whereas elevated S1PR2 expression contributes to the amplification of cutaneous inflammatory responses. Beyond TGR5 and S1PR2 signaling, bile acids can also activate the FXR signaling pathway, which exerts anti-inflammatory effects and maintains intestinal barrier integrity (33). Nevertheless, the precise role and underlying molecular mechanisms of the microbiota-bile acid-FXR axis in AD progression remain to be further elucidated.

Bidirectional metabolic crosstalk between the gut and skin is further exemplified by the local effects of cutaneous inflammation. Within AD lesional skin, a Th2-skewed inflammatory milieu with heightened IL-4 and IL-13 perturbs keratinocyte lipid metabolism. This inhibitory signaling downregulates key metabolic genes (e.g., *FADS2*, *SCD1*), impairs ceramide biosynthesis, and ultimately compromises skin barrier integrity (34). Systemic metabolic disorders such as obesity and diabetes aggravate local skin dysfunction by inducing gut dysbiosis and chronic low-grade inflammation. For example, hyperglycemia promotes the accumulation of advanced glycation end products (AGEs), which bind to keratinocyte RAGE, trigger oxidative stress, and subsequently facilitate *Staphylococcus aureus* colonization (35). In summary, the gut–skin metabolic axis operates through multilayered crosstalk: gut-derived metabolites signal remotely to the skin, systemic metabolic health shapes gut microbial structure, and local cutaneous cytokine milieus govern keratinocyte metabolism and barrier homeostasis.

## 4 Barrier dysfunction and immune polarization in the gut-skin axis

**Figure 1:**
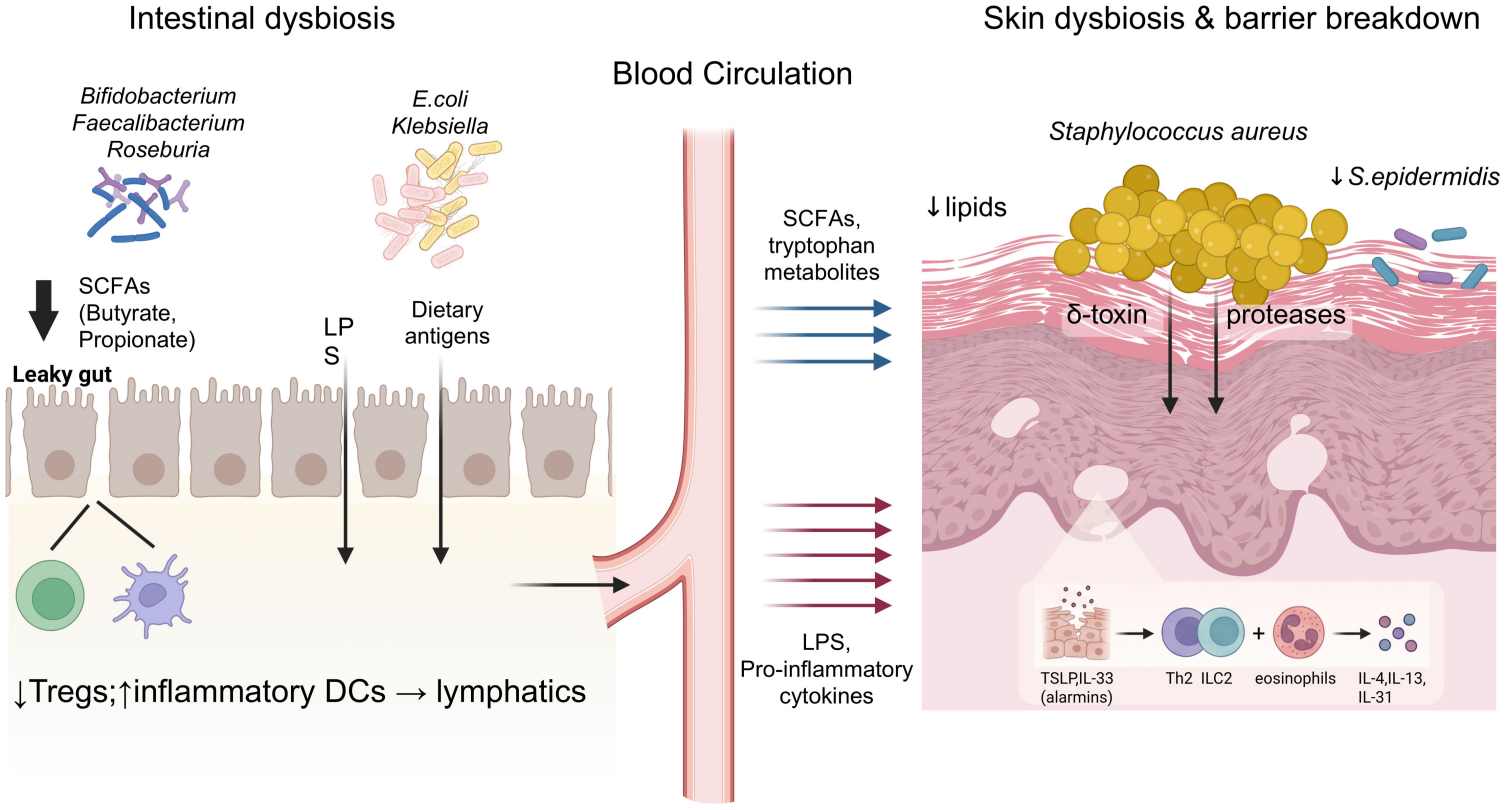
Gut-skin axis dysbiosis in AD. Intestinal dysbiosis in AD reduces beneficial commensals (e.g., *Bifidobacterium*) and increases pathobionts (e.g., *E. coli*), lowering SCFA production and impairing intestinal barrier function. This allows translocation of LPS and dietary antigens, triggering systemic inflammation. Meanwhile, reduced SCFAs and tryptophan metabolites fail to support skin barrier repair. In the skin, *S. aureus* overgrowth and barrier breakdown trigger keratinocyte-derived alarmins (TSLP, IL-33), activating Th2/ILC2 responses to produce type 2 cytokines (IL-4, IL-13, IL-31) and amplify inflammation.

The pathogenesis of AD is fundamentally driven by microbial dysbiosis along the gut-skin axis, a connection that disrupts the integrity of both intestinal and cutaneous barriers, thereby triggering a cascade of innate and adaptive immune dysregulation (**Figure 1**). Specifically, gut microbiota dysbiosis leads to increased intestinal permeability, commonly referred to as “leaky gut”, which permits the translocation of microbial products (e.g., lipopolysaccharides, LPS) into the systemic circulation. This translocation induces a state of chronic low-grade inflammation that progressively impairs the skin barrier (36–38). This inflammatory environment, coupled with direct damage caused by skin pathogens such as *Staphylococcus aureus*, significantly impairs keratinocyte function and disrupts the production of essential barrier proteins (39, 40).

Beyond barrier disruption, microbial components actively engage innate immune receptors, including TLR2, on skin cells, prompting the release of alarmins such as TSLP, IL-33, and IL-25, which are pivotal in initiating and sustaining type 2 inflammation (41). Meanwhile, gut dysbiosis reduces the synthesis of immunoregulatory SCFAs, which are crucial for maintaining the function and stability of Tregs (42, 43). The consequent loss of Treg-mediated suppression effectively eliminates a critical inhibitory mechanism on pro-inflammatory Th2 cells. Collectively, the synergistic interaction between a permeable gut barrier that promotes systemic inflammation and a compromised skin barrier that amplifies local innate immune activation establishes a self-perpetuating vicious cycle. This cycle skews adaptive immunity decisively toward a pathogenic Th2 profile, perpetuating the chronicity of AD.

## 5 Gut-targeted interventions for AD

Accumulating clinical evidence validates the therapeutic and preventive potential of targeted microecological interventions against pediatric AD, including probiotics, prebiotics, and synbiotics. Specifically, well-characterized strains such as *Bifidobacterium animalis* (*B. animalis*) subsp. *lactis* CECT 8145 and *Lactobacillus rhamnosus* (*L. rhamnosus*) GG have been confirmed to alleviate AD symptoms, with their protective effects primarily mediated *via* modulation of the gut–skin axis (44–46). Consistent with the regulatory effects of probiotics, prebiotics and synbiotics also exert favorable immunomodulatory activity and remodel the gut microbiota. Prebiotic substrates such as oligosaccharides and polyphenols can further precisely maintain the homeostasis of the gut–skin axis. Notably, oligosaccharides and polyphenols exert synergistic effects to boost SCFA biosynthesis, which is known to play a protective role in allergic inflammation by promoting regulatory T cell responses and maintaining epithelial barrier integrity (42, 47). Despite these favorable mechanistic observations, cautious extrapolation to routine clinical practice is advised (48–51).

Probiotic, prebiotic and synbiotic interventions have demonstrated clinical efficacy in AD management, capable of markedly lowering the SCORAD index relative to placebo controls (52). Multi-strain combinations and synbiotic formulations tend to yield superior outcomes (53). Nevertheless, the efficacy of these interventions is strictly strain-dependent. For instance, *B. animalis* CECT 8145 enhances SCFA production and modulates gut microbial ecology, whereas *L. rhamnosus* GG reinforces intestinal barrier integrity and promotes Treg/Th17 immune balance, highlighting that strain-specific functional profiles determine therapeutic outcomes in AD (44, 45). The optimal dosage, intervention duration and long-term safety profile still require verification via large-scale, standardized clinical trials (54, 55), and substantial heterogeneity among existing studies hinders the establishment of definitive clinical guidelines (56).

Beyond viable probiotic microorganisms, microbe-derived bioactive products and microbiome-based ecological interventions have emerged as novel strategies to target the gut–skin axis in AD pathogenesis. Postbiotics, defined as non-viable microbial cells, cellular components and metabolic byproducts, represent a promising therapeutic frontier, encompassing bacterial lysates, extracellular polysaccharides, and key metabolites such as SCFAs (35, 57). SCFAs, such as butyrate, acetate, and propionate, are fermentation products of dietary fiber and play crucial roles in maintaining epithelial barrier integrity and sustaining immune homeostasis (57). Preclinical findings showed that topical administration of SCFAs effectively ameliorated cutaneous lesions in murine AD models (35). Clinical observations further indicate that reduced circulating SCFA levels correlate with increased AD severity in humans (58).

FMT constitutes a more radical microbiome intervention designed to reconstruct disrupted gut microbiota. Emerging preliminary evidence supports the clinical benefits of FMT for AD. Current studies have shown significant reductions in AD clinical scores after FMT, accompanied by successful donor microbial engraftment, gut microbiota remodeling and normalized immune responses (59, 60). Comparable therapeutic benefits have also been validated in canine and murine AD models (61–64). As an optimized alternative, washed microbiota transplantation (WMT) has exhibited favorable efficacy and safety, contributing to improved clinical indices and gut microbiota rebalance (65). Even so, FMT carries inherent risks such as pathogen transmission, mandating rigorous donor screening and further safety assessment before widespread clinical adoption (66). In addition, donor variability, long-term engraftment stability, and the lack of standardized protocols remain major challenges that limit the clinical translation of FMT for AD, and current evidence is insufficient to define optimal donor selection or treatment regimens. To mitigate these risks, stringent donor screening including stool testing for enteric pathogens and multidrug-resistant organisms, as well as serological screening for HIV, HBV, and HCV is essential, alongside long-term surveillance of clinical response durability and gut microbiome engraftment stability. In addition, dietary modification serves as a fundamental cornerstone of AD holistic management. High-fiber dietary patterns boost endogenous SCFA synthesis and correlate with reduced AD susceptibility (67). In contrast, a high-fat diet (HFD) aggravates AD-like cutaneous manifestations in mice, a process closely linked to gut microbiota dysbiosis and suppressed SCFA production. Notably, acetate supplementation can reverse HFD-induced exacerbation (6). The Mediterranean diet, rich in vegetables, fruits, legumes, fish, and olive oil, is associated with lower AD incidence and milder disease severity in both offspring and adults, plausibly attributable to its anti-inflammatory properties and modulatory effects on the gut–skin axis.

## 6 Integrated strategies: microbiome-targeted therapies and host-modulating drugs for AD

Personalized medicine stratifies AD patients by microbial profiles. A study using shotgun metagenomic analysis of the skin microbiome from patients with AD and healthy controls identified distinct microbiome configurations (dermotypes A and B), in which dermotype B, characterized by reduced microbial richness and pathogenic *Staphylococcus* abundance, correlates with severe disease and offers prognostic utility (16). Integrated multi-omics approaches combining genomics, transcriptomics, proteomics and metabolomics allow the identification of patient-specific dysbiotic signatures and immune aberrations, laying the foundation for precision targeted therapy (68–71).

Precise and targeted therapeutic strategies for AD primarily aim to rectify skin microbial dysbiosis by eliminating pathogenic taxa such as *S. aureus* (72, 73), with alternatives to antibiotics being sought for MRSA (74, 75). Antimicrobial peptides (AMPs) represent an appealing candidate due to their anti-*S. aureus* and intrinsic immunomodulatory properties (60, 76). Nevertheless, clinical trials of omiganan have demonstrated that AMP monotherapy can remodel the skin microbiome without yielding tangible clinical benefits, implying that single-target intervention alone is inadequate to reverse AD pathogenesis (77).

Bacteriophage therapy enables targeted decolonization of pathogens. Novel phages such as SaGU1 have been shown to reduce *S. aureus* burden in experimental models (78, 79), and their therapeutic efficacy can be further augmented by combination with surfactants (80) or co-administration with skin commensals to mitigate the risk of bacterial resistance development (81). Another favorable strategy leverages beneficial skin commensals. For instance, *Staphylococcus epidermidis* suppresses *S. aureus* overgrowth via secreting antimicrobial substances and regulating cutaneous immune responses (82). Similarly, prebiotic agents such as acacia gum can reshape skin microbial communities and alleviate inflammatory responses in AD experimental models (83). Broadly speaking, current microbial restoration approaches for AD encompass probiotic intervention (84), supplementation of key microbial metabolites such as butyrate to attenuate inflammation (85), and topical regimens that redirect skin flora toward a commensal-dominated state (86). Collectively, these strategies seek to re-establish microbial and immune homeostasis, thereby restraining AD inflammation and repairing skin barrier function.

Beyond direct microbial modulation, targeting immune pathways that sense and interact with the microbiota constitutes an alternative therapeutic paradigm for AD. Dupilumab, an FDA-authorized IL-4Rα inhibitor, exerts robust inhibitory effects on type 2 inflammatory responses and simultaneously remodels skin bacterial communities, manifesting as decreased *S. aureus* abundance and recovered microbial richness (87, 88). In addition, dupilumab remodels cutaneous fungal flora and intestinal microbiome, alongside restored homeostasis of tryptophan metabolism (87, 89, 90). Activated type 2 inflammation is well-documented to inhibits the expression of skin-derived AMPs, which restricts the viability of antibiotic-producing coagulase-negative staphylococci (CoNS-AM+) and in turn indirectly boosts the expansion of *S. aureus* (82). Therefore, the suppression of cutaneous *S. aureus* by dupilumab is likely a consequence of reduced type 2 inflammation rather than a direct mechanism of action.

Novel small molecules further target the host–microbe signaling interface. The AHR is indispensable for maintaining skin barrier integrity and immune homeostasis (26, 27), and the topical AHR agonist Tapinarof has achieved satisfactory therapeutic outcomes in AD clinical trials (26, 28). Skin commensals can also activate AHR signaling to enhance host defense and inhibit *S. aureus* colonization (29, 30). Other immune targets, such as TLR2, provide additional opportunities for fine-tuning microbiota-related immune responses (91). Natural agents such as plant-derived polyphenols and traditional herbal botanicals provide alternative therapeutic approaches. Benefiting from their capacity to act on multiple targets and signaling pathways, natural products represent promising multi-target regimens for the long-term clinical AD management (92). Due to their anti-inflammatory properties, polyphenols have been found to participate in the neutralization of reactive oxygen species (ROS) and sustain immune homeostasis. Meanwhile, they indirectly regulate the gut–skin axis by reshaping intestinal microbial composition and altering the generation of microbial metabolites, thereby modulating cutaneous inflammatory responses (93). Mohammad et al. (94) further demonstrated that genistein and icaritin offer a comprehensive approach against AD by improving barrier integrity and cutaneous hydration while reducing microbial susceptibility. In inflamed keratinocytes, both phytochemicals markedly elevated the expression of barrier-related proteins (FLG, claudin-1, ZO-1), moisturizing mediators (AQP3, HAS1, HAS2), and antimicrobial peptides (hBD3), with superior efficacy compared to dexamethasone. Nevertheless, further formulation refinement and clinical investigation are required to convert these natural bioactive substances into safe and efficacious therapeutic agents for AD patients.

## 7 Conclusion

The gut–skin axis represents a pivotal paradigm shift in the comprehension of AD. It fundamentally redefines intestinal and cutaneous dysbiosis as core, active drivers of AD pathogenesis, rather than passive bystanders of disease progression. Such microbial imbalance initiates a self-sustaining vicious cycle of skin barrier impairment and pervasive immune dysregulation, which is mediated by the intricate crosstalk of immune, metabolic and neuroendocrine signaling pathways. Accordingly, a holistic and rigorous interpretation of AD requires multidisciplinary integration spanning gastroenterology, dermatology and immunology. AD should be recognized as a pathological state arising from systemic network instability, instead of being attributed to a single microbial strain or isolated metabolite.

This integrated conceptual framework lays a solid theoretical foundation for developing innovative therapeutic modalities targeting the gut–skin axis, encompassing interventions such as supplementation with strain-specific probiotics, prebiotics and postbiotics, FMT, and individualized dietary regulation (**Table 1**). While mechanistic investigations have yielded encouraging theoretical evidence to validate these therapeutic approaches, current clinical data exhibit substantial heterogeneity, constrained by inherent methodological drawbacks including limited sample sizes, brief intervention durations, and non-uniform preparation specifications. Furthermore, the present narrative review is susceptible to literature selection bias, and all conclusions drawn herein are subject to the authors’ subjective interpretation of published studies. Therefore, the clinical application of gut–skin axis-targeted therapies call for cautious optimism, which must be grounded in rigorous, well-designed and reproducible clinical trials to clarify the efficacy of each intervention and identify its applicable patient subgroups.

**Table 1:** Key gut–skin axis-targeted therapeutic strategies for atopic dermatitis.

Therapeutic target	Intervention type	Specific examples/agents	Key mechanisms in the gut-skin axis	Current application & clinical effects
Gut microbiome modulation *(Section 5)*	Probiotics	*Bifidobacterium animalis* subsp *lactis* CECT 8145, *L. rhamnosus* GG	Expand Treg populations, suppress pro-inflammatory cytokines.	Clinical: significantly lowers SCORAD index; efficacious in pediatric AD.
	Prebiotics & synbiotics	Dietary fiber, multi-strain synbiotics	Stimulate growth of beneficial flora, boost endogenous SCFA synthesis.	Clinical: exerts immunomodulatory effects; strain-and dose-dependent efficacy.
	Postbiotics	SCFAs (butyrate, acetate, propionate)	Maintain intestinal epithelial barrier integrity, exert systemic anti-inflammatory effects.	Preclinical/clinical: topical application ameliorates skin lesions; reverses HFD-induced exacerbation.
	FMT/WMT	Donor fecal microbiota, washed microbiota	Reconstruct disrupted gut ecosystems, restore systemic immune and metabolic homeostasis.	Clinical: significant reductions in AD clinical scores; restores gut microbiota balance.
	Dietary adjustments	High-fiber diet, Mediterranean diet	Boost SCFA synthesis, promote anti-inflammatory milieus.	Observational clinical: associated with lower AD incidence and milder severity.
Skin microbiome modulation *(Section 6)*	Pathogen decolonization	Bacteriophages (e.g., SaGU1), antimicrobial peptides (AMPs, e.g., Omiganan)	Targeted elimination of pathogenic *S. aureus*, reducing barrier disruption and toxin release.	Preclinical/Trial: reduces *S. aureus* colonization; AMP monotherapy shows limited clinical benefit.
	Commensal restoration	*Staphylococcus epidermidis*, acacia gum (prebiotic)	Secrete antimicrobial substances to inhibit *S. aureus*, reshape cutaneous immune response.	Preclinical: alleviates local inflammatory responses and restrains *S. aureus* overgrowth.
Host immune modulation *(Section 6)*	Biologics	Dupilumab (IL-4Rα antagonist)	Suppress type 2 inflammation; concurrently remodel both skin and gut microbiome; improve tryptophan metabolism.	Clinical (approved): Potently resolves AD symptoms; restores microbial diversity on the skin.
	Small molecules	Tapinarof (topical AHR agonist)	Activate AHR signaling to enhance host defense and maintain skin barrier integrity.	Clinical (approved/trials): achieves satisfactory therapeutic outcomes; limits *S. aureus* colonization.

Moving forward, the research field is undergoing a transition from generalized one-size-fits-all interventions toward precision medicine. Future therapeutic development will focus on sophisticated combinatorial and individualized strategies. This approach involves meticulously characterizing an individual’s unique microbial and immune fingerprint to guide tailored therapy, potentially pairing established biologics with precisely tailored microbiome modulators to achieve synergistic therapeutic effects. Nevertheless, these combinatorial regimens entail unpredictable safety hazards, such as aberrant systemic immune responses, altered drug metabolism or elevated infection susceptibility among immunocompromised populations. Rigorous risk assessment is therefore indispensable in subsequent research validations. Meanwhile, the development of novel pharmacological agents that target keystone microbes or critical host pathways, such as SCFA or bile acid receptors, represents the exciting next frontier in therapeutic innovation.

Ultimately, the successful translation of the gut-skin axis concept into tangible clinical reality hinges critically on two foundational pillars: first, achieving deep mechanistic insight into the intricate crosstalk between the host and the microbiome, and second, securing robust validation through large-scale longitudinal cohort studies and randomized controlled trials (RCTs). Adhering to this dual research orientation will not only advance the mechanistic understanding of AD, but also facilitate the creation of a new generation of mechanism-driven therapies that target the underlying systemic pathogenesis of this prevalent and complex inflammatory skin disease.
